# Cellular memory after cancer therapy: persistence after drug withdrawal, maintenance across cell divisions, and responses to rechallenge

**DOI:** 10.3389/fphar.2026.1966584

**Published:** 2026-10-05

**Authors:** Chengyan Shi, Ruikun Jiang, Jun Yan

**Affiliations:** The Second Department of Oncology, The First Affiliated Hospital of Heilongjiang University of Chinese Medicine, Harbin, Heilongjiang, China

**Keywords:** cell-state plasticity, drug withdrawal, drug-tolerant persister, minimal residual disease, non-genetic resistance, post-treatment cellular memory, rechallenge

## Abstract

A fraction of tumor cells can survive therapy without acquiring stable resistance mutations and enter a drug-tolerant persister (DTP) state. After drug withdrawal, these cells may resume proliferation and regain drug sensitivity, retain phenotypic features that alter survival or death upon retreatment, or continue to evolve toward stable resistance. The DTP designation describes survival under therapeutic pressure but does not establish whether the associated state persists after treatment ends. The term post-treatment cellular memory is used here operationally for a treatment-associated state that remains detectable after adequate drug washout and can influence a later response. Evidence can be evaluated across three experimental dimensions: persistence after withdrawal, maintenance across subsequent cell divisions or clonal descendants, and a functional consequence upon re-exposure. These dimensions indicate different strengths of support rather than mandatory diagnostic criteria. The review therefore organizes representative studies by what was measured after withdrawal rather than by DTP markers or mechanisms alone. Recent work indicates that AP-1 can contribute to encoding treatment-associated transcriptional states and that CBP/p300 activity can help maintain an established reporter state, including after drug withdrawal; however, molecular persistence does not substitute for demonstrating altered function upon rechallenge with the original agent. Chromatin remodeling, transcriptional state transitions, antioxidant metabolism, autophagy, organelle homeostasis, and lineage plasticity may prolong residual-cell survival. Their biological consequences differ: some states decay after withdrawal, some create time-limited vulnerabilities to ferroptosis or to therapeutic targeting of anti-apoptotic dependencies, and others extend the interval during which genetic resistance can emerge. Lineage tracing, single-cell multi-omics, withdrawal-rechallenge experiments, and longitudinal sampling of minimal residual disease can help distinguish transient stress responses from persistent post-treatment states. These observations provide a rationale for testing whether treatment sequence and interventions during residual disease can be matched to the reversibility and vulnerabilities of residual states.

## 1 Introduction

Radiographic response to targeted therapy or chemotherapy does not indicate that every tumor cell has been eliminated. A small residual population may survive continued treatment, subsequently resume proliferation, and seed recurrent disease. Genetic heterogeneity explains part of this behavior but not the full range of residual-cell states. Sharma et al. identified a small, reversible DTP population across several cancer cell lines. In PC9 cells, these cells accounted for approximately 0.3% of the starting population, showed a reduction in drug sensitivity of more than two orders of magnitude, and exhibited IGF-1R signaling and KDM5A-associated chromatin changes (Sharma et al., 2010). Non-genetic adaptation, transient pre-resistant transcriptional states, and treatment-induced cellular reprogramming have since been observed in other models (Pisco et al., 2013; Shaffer et al., 2017).

DTP research explains how tumor cells can survive intense therapeutic pressure transiently, yet experimental definitions of DTPs vary widely. Drug dose, exposure duration, withdrawal interval, and functional readouts are not standardized; cells surviving 3 days of continuous treatment and cells capable of expansion after several weeks of treatment are sometimes placed in the same category (Russo et al., 2024). This variation directly affects mechanistic interpretation. Both populations may appear to be DTPs during treatment, but after withdrawal one may regain sensitivity whereas the other may retain a state that influences subsequent therapy. Neither the DTP label nor a single epigenetic marker can predict this distinction.

Across the literature, the DTP label is most consistently used for a reversible, non-genetic survival state under ongoing therapy, whereas therapy-persistent residual disease is the translational setting in which those cells can be evaluated before relapse. Neither term, by itself, demonstrates persistence after withdrawal or predicts a functional response to later treatment (Russo et al., 2024).

When a treatment-induced change remains detectable after the drug has been removed and modifies cell death, proliferation, or differentiation during a later exposure, the treatment history continues to shape cellular behavior. Such an effect may decay over time and does not require permanent resistance. Maintenance across cell divisions, altered responses to retreatment, and loss of these differences after perturbation of a candidate mechanism can further distinguish a transient stress response from a persistent post-treatment state. These features are compared in Table 1 and placed on a common temporal trajectory in Figure 1A.

**TABLE 1:** Operational distinctions among acute treatment-induced stress responses, drug-tolerant persister states, post-treatment cellular memory, stable non-genetic resistance, and genetically mediated resistance.

Concept	Operational definition	Status after drug withdrawal	Maintenance across cell divisions	Rechallenge phenotype	Genetic basis	Evidence informative for classification	Principal limitation
Acute treatment-induced stress response	A molecular or phenotypic response induced during or immediately after treatment that rapidly decays after drug removal and does not produce a demonstrable persistent functional effect	Brief or absent after adequate washout	Not demonstrated	No persistent alteration is expected after recovery; this should be confirmed rather than assumed	Not required	Measurement during treatment or shortly after withdrawal, together with an adequate washout and a time-matched untreated control	A transient marker increase may be misclassified as a persistent state when withdrawal and functional testing are omitted
Drug-tolerant persister state	A residual population that survives an otherwise lethal or strongly inhibitory treatment without requiring a stable resistance mutation	Not established by the DTP designation	Not required to identify survival under treatment; assessment after withdrawal is a separate experimental dimension	Variable; sensitivity may recover after withdrawal	No stable resistance mutation is required	Survival under a defined drug concentration, exposure duration, and residual-cell endpoint	The DTP label describes survival under treatment and cannot by itself distinguish a transient state from post-treatment cellular memory
Post-treatment cellular memory (operational use)	A treatment-associated molecular or phenotypic state that remains detectable after drug withdrawal and can influence survival, proliferation, differentiation, or cell death during a later exposure	Present for a measurable interval after adequate washout	One experimental dimension. Persistence across several divisions or clonal descendants provides stronger evidence of mitotic maintenance, but no universal threshold exists	Altered relative to treatment-naive or time- and passage-matched controls	No stable explanatory resistance mutation	Evidence can be compared across adequate washout, repeated post-withdrawal measurements, maintenance across divisions or descendants, and functional rechallenge. Lineage tracing, genomic assessment, and perturbation or rescue strengthen interpretation; the dimensions are not a validated mandatory checklist	No universal threshold defines the required duration or number of divisions, and residual drug or clonal selection can mimic persistence
Stable non-genetic resistance	A long-lasting resistant phenotype that remains stable under drug-free conditions without an identified genetic alteration sufficient to explain resistance	Prolonged	Yes; stable through repeated passages	Persistent reduction in sensitivity or resistance	No identified explanatory alteration	Long-term drug-free stability, repeated rechallenge, and sufficiently comprehensive genomic assessment	Undetected genetic alterations, copy-number changes, or selection of a minor clone may be misclassified as non-genetic resistance
Genetically mediated stable resistance	A stable resistant phenotype driven or strongly supported by an acquired or selected genetic alteration, including expansion of a pre-existing mutant clone or acquisition of a resistance alteration during residual-cell survival	Stable	Stable and heritable	Persistent resistance	Present or strongly implicated	Identification of the resistance alteration together with functional validation or longitudinal clonal evidence	The timing of mutation acquisition and the contribution of preceding non-genetic tolerance may remain unresolved
Reversible residual-tumor state after treatment cessation	A treatment-associated state detected in residual tumors that resolves as the tumor regrows after treatment cessation, with recovery of treatment sensitivity and baseline-like molecular or histologic features	Decays during regrowth after treatment cessation	Continuous maintenance is not established; reversal argues against stable propagation of the bulk residual state	Recovered sensitivity can be demonstrated in regrown tumors	Major clonal selection is not required, but clonal architecture should be assessed	Paired baseline, residual, and regrown samples; treatment-response testing after regrowth; and genomic or barcode assessment of clonal architecture. (Echeverria et al., 2019)	Recovery of a bulk treatment-naive-like state does not exclude persistent rare subpopulations and does not demonstrate continuous molecular memory during the drug-free interval
Time-dependent transition from early tolerance to late persistence during treatment	Sequential surviving states that arise at different times during ongoing treatment and use different stress-response, repair, or survival programs	Not established unless a separate withdrawal phase is tested	Should be measured rather than inferred from elapsed exposure time	Not established by time-resolved measurements made only during treatment	No stable resistance alteration is required for the transition	Multiple exposure time points with matched viability, clonal, and molecular measurements that distinguish early and late surviving populations. (Punzi et al., 2025)	A transition during continued exposure defines temporal heterogeneity in tolerance or persistence, not post-treatment cellular memory after withdrawal

Definitions are operational and do not establish universal biological thresholds. Evidence can be compared across three experimental dimensions—persistence after adequate withdrawal or washout, maintenance across cell divisions or clonal descendants, and functional consequence upon rechallenge. These dimensions are complementary indicators of evidentiary strength rather than mandatory diagnostic criteria.

Abbreviations: DTP, drug-tolerant persister.

**FIGURE 1:**
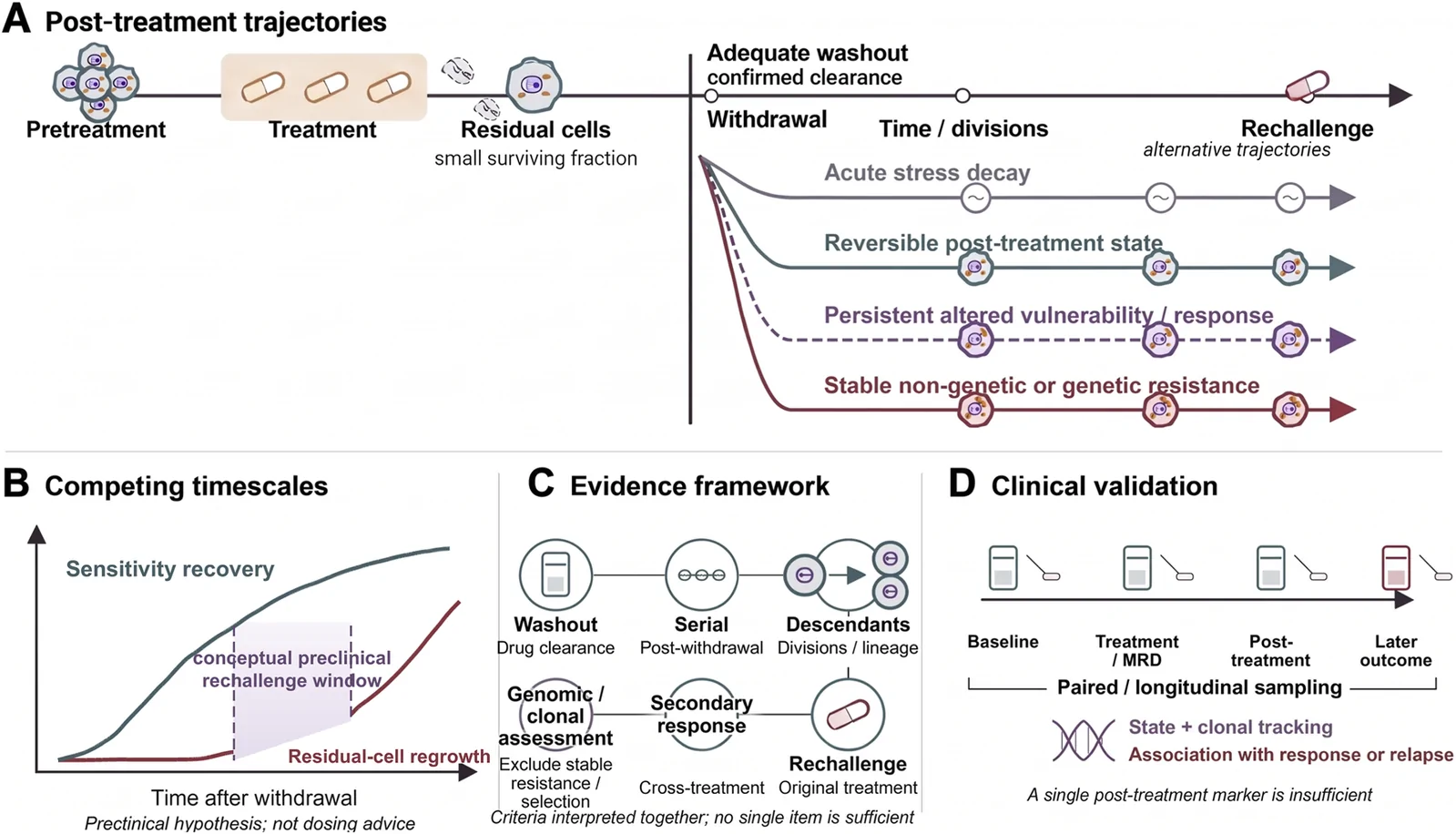
Post-withdrawal trajectories, evidentiary requirements, and translational interpretation **(A)** Residual-cell survival during treatment is the starting point, not evidence of post-treatment memory. After adequate drug washout and confirmation of pharmacologic clearance, residual cells may show rapid resolution of an acute stress response, a reversible state retained for a limited interval, a persistent secondary vulnerability or altered response, or stable non-genetic or genetically mediated resistance **(B)** Sensitivity recovery and residual-cell regrowth may proceed at different rates. A preclinical scheduling hypothesis can be tested when sensitivity to the original treatment recovers before substantial residual-cell expansion; the conceptual curves are not drawn to scale and do not support routine treatment interruption or rechallenge **(C)** Informative designs combine repeated post-withdrawal measurements, assessment across cell divisions or clonal descendants, original-treatment rechallenge, testing of secondary or cross-treatment responses, and genomic or clonal analysis to exclude stable resistance and selection **(D)** Clinical validation requires longitudinal sampling from baseline through treatment and minimal residual disease, confirmation of drug clearance after treatment completion or a clinically indicated interruption, joint state and clonal characterization, and association with a later response or relapse. Treatment should not be withheld solely to create a washout interval.

The objective of this narrative review is to determine how current experimental evidence supports, or fails to support, the retention of treatment-associated cell states after drug removal. We focus on tumor-cell and microenvironmental mechanisms relevant to state formation, persistence, decay, and later response, and on the designs needed to distinguish these possibilities. The scope includes targeted therapy, chemotherapy, immunotherapy-persistent states, residual disease, and regrowth models when they inform withdrawal or subsequent treatment; on-treatment survival alone is not treated as cellular memory. Clarifying these boundaries is important because hypotheses about residual-disease intervention, treatment sequence, and rechallenge depend on whether the relevant state outlasts pharmacologic exposure.

Recent reviews have synthesized DTP biology from different perspectives. Russo et al. connected biological questions to clinical opportunities (Russo et al., 2024). Li et al. emphasized reversibility, microenvironmental interactions, and intervention strategies (Li et al., 2025). Liu et al. integrated DTP generation under targeted therapy or immunotherapy with tumor-cell, intercellular, host-ecology, and clinical perspectives (Liu et al., 2025); and Lopez-Gonzalez et al. systematically mapped definitions, experimental models, molecular mechanisms, and post-treatment recovery across 343 eligible records Lopez-Gonzalez et al. (2026). Additional reviews have addressed targeted-therapy-tolerant persisters, therapy-persistent residual disease, and persister plasticity (Cabanos and Hata, 2021; McDonald and Dedhar, 2024; Sun et al., 2024). The present review addresses a narrower evidentiary question: after the initiating drug has been adequately cleared, what observations are required to show that prior treatment has left a state that remains detectable and changes a later response? We therefore compare representative studies according to post-withdrawal persistence, maintenance across divisions or clonal descendants, and functional rechallenge, while explicitly separating state retention from residual drug, clonal selection, stable non-genetic resistance, and genetically mediated resistance.

### 1.1 Literature identification

PubMed was searched through 7 September 2026 using combinations of terms related to drug-tolerant persisters, residual disease, non-genetic resistance, cellular, epigenetic, transcriptional, and metabolic memory, drug withdrawal, washout, rechallenge, lineage tracing, single-cell analysis, and the tumor microenvironment.

The foundational searches were supplemented by backward and forward citation tracking; Crossref and OpenAlex were used only as supplementary bibliographic discovery tools. Because this is a narrative review, studies were selected for their relevance to distinctions among on-treatment persistence, withdrawal, division-linked maintenance, rechallenge, and stable resistance; no formal risk-of-bias assessment or exhaustive screening flow was performed.

## 2 Establishing persistence after treatment: drug withdrawal, passaging, and rechallenge

Survival during treatment demonstrates tolerance only under the conditions present at that time. Sharma et al. found that DTPs regrown after drug withdrawal regained sensitivity to the original agent, indicating that at least some tolerant states decay once therapeutic pressure is removed (Sharma et al., 2010). Evidence for post-treatment persistence is strongest when molecular or phenotypic differences remain across subsequent cell divisions or clonal descendants and alter survival, death, or proliferation upon retreatment. Requirements for withdrawal intervals and tests of reversibility remain inconsistent across DTP studies, making comparisons between models difficult (Russo et al., 2024; Lopez-Gonzalez et al., 2026). Stable mutations or long-lasting irreversible epigenetic changes can instead fix resistance, with a biological basis distinct from that of a post-treatment state that can decay (Hata et al., 2016; Shaffer et al., 2017).

The duration of observation should reflect the doubling time of the tumor model and the treatment used; a single withdrawal interval expressed in days is unlikely to suit every setting (Russo et al., 2024; Lopez-Gonzalez et al., 2026). Detection of the same transcriptional feature after treatment may reflect continued expansion of an original clone or convergence of several clones on a similar state. Barcode-tracing studies in colorectal and breast cancer models have shown that clonal origin, proliferative status, and transcriptional programs can change concurrently within residual populations (Oren et al., 2021; Rehman et al., 2021). Untreated cells carried through the same culture and passaging schedule can help exclude state drift caused by prolonged culture, while cell-count changes after retreatment should be resolved into reduced proliferation and cell death. Rechallenge should also extend beyond the original agent because, in specific models, drug-tolerant and immunotherapy-persistent cells exhibit tolerance across treatment modalities (Davern et al., 2026).

Reducing DTP abundance during treatment alone does not establish that a chromatin or metabolic mechanism sustains the post-treatment state. After lapatinib withdrawal, regrown cells retained sensitivity to GPX4 inhibition for at least 2 weeks before this vulnerability gradually diminished, showing that a post-treatment vulnerability can itself change over time (Hangauer et al., 2017). A mechanism is more strongly implicated in persistence when its perturbation accelerates phenotypic decay during withdrawal and restores sensitivity upon rechallenge. Residual drug, differences in proliferation, and expansion of a minor clone can produce similar observations and should be assessed through washout, cell-death measurements, and lineage information.

Direct withdrawal-regrowth evidence is available in triple-negative breast cancer patient-derived xenografts. Residual tumors after doxorubicin plus cyclophosphamide retained the subclonal architecture of untreated tumors but displayed distinct molecular and histologic states; after treatment cessation, they regrew as chemotherapy-sensitive tumors resembling untreated controls. This supports a reversible, largely nonselective tolerant state, but it does not establish that a specific molecular program remained functionally active throughout the drug-free interval (Echeverria et al., 2019).

Time-resolved chemotherapy models have further separated an early autophagy-associated tolerant response from later persistence accompanied by reversal of the initial DNA-repair program. These findings indicate that short and prolonged survival endpoints can represent biologically distinct responses and should not be treated as interchangeable definitions of persistence (Punzi et al., 2025).

## 3 Formation and persistence of cell states under therapeutic pressure

### 3.1 Pre-existing cell states and treatment-induced reprogramming

Cell populations are not uniform before treatment. In melanoma, a small subset of cells transiently expresses high levels of resistance-associated genes. Although these cells do not initially possess stable resistance, they are more likely to survive drug exposure and undergo further epigenetic reprogramming, eventually acquiring a stable resistant phenotype (Shaffer et al., 2017). Transient variation before treatment can therefore influence early survival, after which drug exposure further reshapes the surviving cells.

Other models indicate that entry into a DTP state is not confined to a few predetermined clones. Under chemotherapy, colorectal cancer cells can adopt an embryonic diapause-like state. Recurrent tumors retain substantial barcode complexity after passing through the DTP phase, suggesting that many clones can enter this state. During this phase, cells reduce proliferation and depend on autophagy for survival (Rehman et al., 2021). Pre-treatment states and treatment-induced changes therefore often contribute together to DTP formation, with their relative importance varying by cancer type, drug, and exposure duration.

### 3.2 Encoding treatment experience in chromatin and transcriptional states

Mechanisms that facilitate entry into or survival of a DTP state during treatment should be distinguished from mechanisms that preserve a treatment-associated state after drug withdrawal. The reversibility of DTPs places their stability between an immediate signaling response and a permanent genetic alteration. Sharma et al. found that the DTP state depends on KDM5A and associated chromatin changes, and that individual cells can enter and exit this state at low frequency (Sharma et al., 2010). In melanoma, cells with transient expression of resistance genes also undergo more persistent transcriptional and chromatin reprogramming during drug exposure (Shaffer et al., 2017). These findings implicate chromatin regulation in DTP formation and state transition, but they do not by themselves establish maintenance after withdrawal.

A trametinib model further showed that treatment history can redirect the fate of a pre-existing transcriptional state. Cells were briefly exposed to dexamethasone, after which dexamethasone was withdrawn and trametinib was introduced; 1 month later, the genes induced at the outset remained more highly expressed. Dual-reporter systems and live-cell tracking also showed that stochastic expression differences present at treatment initiation could be retained in resistant clones. AP-1 inhibition during low-dose exposure reduced subsequent tolerance to high-dose treatment, whereas maintenance of an established reporter-gene state depended on the acetyltransferase activity and bromodomain of CBP/p300 (Li et al., 2026). These experiments connect the expression state at treatment initiation with resistant-clone descendants and suggest that state formation and maintenance may depend on partially distinct mechanisms. Separate reporter experiments also showed that an established AP-1 reporter state remained detectable during 3 weeks of drug-free follow-up after 2 weeks of trametinib exposure. However, the study did not test whether the retained state altered the response when trametinib was reintroduced; it therefore supports molecular persistence after withdrawal rather than a complete functional withdrawal–rechallenge demonstration.

Enhancer remodeling, altered chromatin accessibility, transcription-factor switching, and RNA regulation have all been implicated in DTP survival (Russo et al., 2024; Lopez-Gonzalez et al., 2026). These changes often occur as interacting networks. Their relevance to post-treatment memory depends on whether specific combinations persist after drug withdrawal and predict the response to rechallenge. Figures 2A,B separate mechanisms supporting treatment-period formation or survival from candidate mechanisms of post-withdrawal maintenance.

**FIGURE 2:**
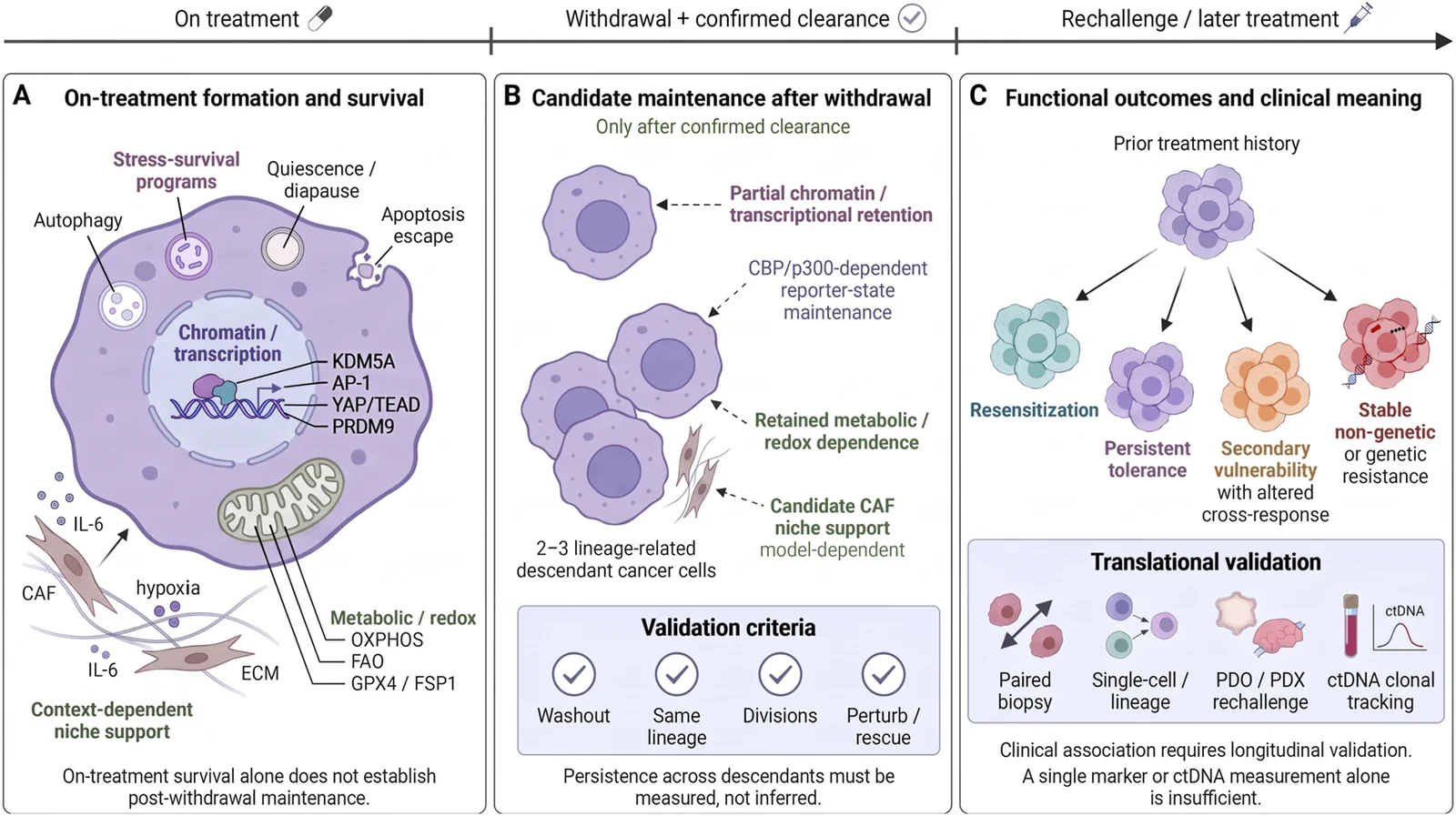
Stage-specific mechanisms and functional outcomes of residual-cell states **(A)** During treatment, DTP formation and survival can involve KDM5A-associated chromatin regulation, AP-1 adaptation, YAP-TEAD or PRDM9-dependent transcriptional programs, quiescence or diapause-like states, apoptosis escape, autophagy, OXPHOS or fatty-acid oxidation, GPX4/FSP1-linked redox defense, and model-dependent stromal support. These mechanisms need not coexist and do not by themselves demonstrate a state after withdrawal **(B)** After confirmed drug clearance, candidate maintenance mechanisms include partial chromatin or transcriptional retention, CBP/p300-associated maintenance in the reporter model, retained metabolic or redox dependencies, and model-dependent niche support, including candidate CAF-associated inputs. Dashed arrows indicate incomplete or context-dependent cross-stage evidence. Maintenance across cell divisions or clonal descendants must be measured rather than inferred **(C)** Functional outcomes after prior treatment include resensitization, persistent tolerance, secondary vulnerability or altered cross-response, and stable non-genetic or genetic resistance. Translational validation can combine paired longitudinal biopsies, single-cell or lineage analysis, PDO/PDX rechallenge, and ctDNA-based clonal tracking. Clinical interpretation requires longitudinal association with later response or relapse; a single post-treatment marker or ctDNA measurement alone is insufficient to establish a persistent treatment-associated state.

In EGFR-mutant lung cancer, combined EGFR and MEK inhibition can uncover a senescence-like dormant population in which YAP/TEAD activity engages SLUG and represses the pro-apoptotic factor BMF. Independent genome-wide CRISPR screens also identified Hippo-pathway regulators as determinants of osimertinib or gefitinib persistence, while patient-derived and immunocompetent models implicated a FAK-YAP signaling module across EGFR-, ALK-, and KRAS-directed therapies. These studies establish model-specific survival mechanisms during treatment; none alone demonstrates that the same state persists after withdrawal and alters rechallenge response (Kurppa et al., 2020; Haderk et al., 2024; Pfeifer et al., 2024).

Additional studies illustrate that chromatin control is not reducible to one regulator. PRDM9-dependent H3K4me3 supported cholesterol-biosynthesis programs and chemotherapy tolerance in glioblastoma stem-cell models, whereas patient-derived triple-negative breast cancer models converged on basal-keratin, stress, and inflammatory programs regulated in part by FOSL1/AP-1. These mechanisms broaden Figure 2 but remain context dependent and should not be presented as universal maintenance machinery (Baudre et al., 2025; Joun et al., 2025).

### 3.3 Metabolic, autophagic, and lineage heterogeneity among DTPs

DTPs do not merely stop dividing. Residual cells under therapeutic pressure must reorganize energy supply, redox balance, and organelle quality control. Persisters from multiple cancer lineages show heightened sensitivity to GPX4 inhibition and ferroptosis, suggesting that protection against lipid peroxidation is a survival dependency in at least some DTPs (Hangauer et al., 2017). FZD7-marked cells in platinum-tolerant ovarian cancer are likewise vulnerable to ferroptosis (Wang et al., 2021). Diapause-like DTPs in colorectal cancer instead depend on autophagy to recycle intracellular components (Rehman et al., 2021). Persistent metabolic changes in residual breast cancer lesions further indicate that metabolic states can remain for a period after treatment ends (Radic Shechter et al., 2021).

Residual populations are themselves heterogeneous (Rambow et al., 2018; Russo et al., 2024). Using Watermelon barcoding to record clonal origin, proliferative status, and transcriptional features simultaneously, Oren et al. found that proliferative and non-proliferative persisters arose from different lineages. Proliferative persisters had stronger antioxidant programs and made greater use of fatty acid oxidation (Oren et al., 2021). Minimal residual disease in melanoma also contains several interconvertible states. RXR signaling contributes to the formation of a neural crest-like state, and its inhibition reduces accumulation of these cells and delays resistance (Rambow et al., 2018). Cells that persist after treatment therefore comprise several groups with distinct proliferative capacities and metabolic dependencies.

Functional studies likewise identify nonoverlapping vulnerabilities among residual populations. ALDH1A3-mTOR signaling supported chemotherapy-tolerant gastric cancer cells; FSP1-linked lipid and iron homeostasis was associated with cisplatin-tolerant head and neck cancer models; BET inhibition preferentially depleted persisters that had re-entered the cell cycle and delayed relapse in melanoma and lung cancer models; and PRMT1 loss reduced persistence during EGFR- or KRASG12C-targeted therapy in selected lung cancer systems. These observations support state-matched intervention rather than a single pan-DTP target (Kawakami et al., 2020; Chen et al., 2024; Wu et al., 2024; Sun et al., 2025).

These nonoverlapping dependencies complicate combination design (Rambow et al., 2018; Kawakami et al., 2020; Oren et al., 2021; Rehman et al., 2021; Chen et al., 2024; Wu et al., 2024; Sun et al., 2025). A single pan-DTP target could spare states sustained by a different program, whereas simultaneous treatment would be justified only if the vulnerable states coexist in the same model and at the same time. Sequential treatment may be more appropriate when persister states emerge or interconvert over time. Before either strategy is advanced, relevant models should establish same-model coexistence, state-transition kinetics, pharmacologic antagonism, and combined toxicity. At present, these considerations provide a preclinical rationale for state-informed combination experiments rather than a clinical treatment recommendation.

### 3.4 State decay after drug withdrawal and cross-treatment tolerance

Drug-withdrawal experiments can directly show whether a DTP state decays. Sharma et al. observed that cells regrown after withdrawal regained sensitivity to the original agent (Sharma et al., 2010). This recovery indicates that at least some DTPs do not become permanently resistant. Certain post-treatment vulnerabilities can persist longer. Following lapatinib withdrawal, regrown cells remained sensitive to the GPX4 inhibitor RSL3 for at least 2 weeks, after which this sensitivity gradually declined over a longer interval (Hangauer et al., 2017). Recovery of sensitivity to the original drug and loss of a newly acquired vulnerability may therefore proceed at different rates.

Persistent changes after one treatment may also influence the response to another. In experimental models, both drug-tolerant and immunotherapy-persistent cells showed reduced sensitivity to mitochondrial apoptosis, and some showed cross-tolerance to radiation, drugs, or CAR-T-cell attack (Davern et al., 2026). This evidence currently derives mainly from selected cell and animal models and cannot establish that cross-treatment tolerance is universal. It nevertheless indicates that rechallenge experiments should not be restricted to the original agent.

### 3.5 DTP survival extends the interval in which genetic resistance can emerge

In EGFR-mutant lung cancer, T790M resistance can emerge through two distinct courses. T790M-positive clones present before treatment can expand early, whereas DTPs initially lacking T790M may survive long enough to acquire the mutation and develop stable resistance later (Hata et al., 2016). A similar continuum occurs in melanoma: a transient pre-resistant transcriptional state first increases the probability of survival, followed by treatment-induced reprogramming that progressively stabilizes resistance (Shaffer et al., 2017).

These findings do not show that non-genetic tolerance directly induces a specific mutation. DTPs can extend survival under therapeutic pressure, allowing time for subsequent diversification, clonal expansion, and selection. A phenotype that remains stable in drug-free culture without an identified explanatory genetic alteration should not be conflated with genetically mediated resistance carrying changes such as T790M. Pre-existing resistant clones can be selected and expand soon after therapy begins, providing a rationale for studying baseline or early detection and upfront suppression when a targetable alteration is present. By contrast, DTP-derived resistance requires survival, diversification, and selection over a longer residual-disease interval, providing a rationale for testing interventions against the persister reservoir or its evolutionary capacity (Hata et al., 2016; Ramirez et al., 2016; Kashima et al., 2021; Garcia et al., 2025). These distinctions generate testable scheduling hypotheses rather than established dosing rules, and neither route can be inferred from a single post-progression sample.

Persister-derived resistance is also heterogeneous. Erlotinib-resistant colonies descending from a single EGFR-addicted persister acquired multiple clinically recognized resistance mechanisms, and single-cell transcriptomic and chromatin analyses identified divergent tolerant-to-resistant trajectories that included CD74-associated survival. More recently, APOBEC3 induction during EGFR inhibition was shown to promote persister survival and to alter the probability of T790M acquisition and squamous transdifferentiation. These studies support a reservoir model of evolution, but they do not imply that every persister acquires mutations or that one tolerant state determines a unique resistance genotype (Ramirez et al., 2016; Kashima et al., 2021; Garcia et al., 2025).

## 4 Distinguishing clonal selection from state transition through lineage tracing and longitudinal sampling

A transcriptome measured at a single time point describes only the state at sampling and cannot determine whether that state arose through selection of a pre-treatment clone or a treatment-induced cellular transition. Barcodes, heritable tracers, and clonal tracking can record cellular origin, while single-cell transcriptomics, chromatin accessibility, proteomics, and metabolomics can compare the same lineage before treatment, during exposure, after withdrawal, and following rechallenge (Rambow et al., 2018; Oren et al., 2021; Rehman et al., 2021). Dual-reporter live-cell tracking can further link expression differences at treatment initiation to individual resistant clones, testing whether the initial state is retained in their descendants (Li et al., 2026). Together, these approaches can separate changes in clone abundance from state changes within a clone.

Experimental reports should specify drug dose, exposure duration, withdrawal interval, number of cell divisions, and rechallenge conditions. Functional endpoints should extend beyond the surviving fraction to include the mode of cell death, clonal expansion, differentiation trajectory, and dose response upon retreatment. Inhibition, restoration, and rescue of a candidate maintenance mechanism can further distinguish a role in survival during treatment from a role in state persistence after withdrawal. Figure 1C summarizes the design features needed to discriminate state retention from residual drug, clonal selection, and stable resistance.

Longitudinal information is more difficult to obtain from clinical samples. An informative design would pair pretreatment tissue with samples collected during early therapy, minimal residual disease at radiographic response, and recurrent disease. Melanoma studies have shown that minimal residual disease contains multiple cell states and displays spatial heterogeneity (Rambow et al., 2018). Pan-cancer transcriptomic analyses have also identified residual-disease programs shared across several tumor models after chemotherapy (Liu et al., 2024). Lineage and clonal tracking remain necessary to distinguish selection from within-clone state change. Translational approaches for identifying these states in patients are discussed in Section 6. Representative studies are summarized in Table 2, with an extended evidence matrix in Supplementary Table S1.

**TABLE 2:** Representative experimental studies compared across treatment, withdrawal, division-linked maintenance, and rechallenge dimensions.

Study	Cancer model/treatment	On-treatment persistence	Defined withdrawal/​washout	Post-withdrawal persistence	Divisions/clonal descendants	Original-agent rechallenge	Secondary/cross-treatment response	Lineage/single-cell evidence	Evidence interpretation
(Sharma et al., 2010)	Multiple lines; PC9 EGFR-mutant lung cancer; targeted therapy	Yes — rare reversible DTPs survived	Yes — drug removed; regrowth assessed	Yes — tolerance decayed after withdrawal	Partial — regrowth shown; divisions not quantified	Yes — regrown cells recovered sensitivity	Not central	Partial — state entry/exit observed; no lineage recorder	Survival during treatment is not stable memory; reversibility was demonstrated
(Hata et al., 2016)	EGFR-mutant lung cancer; EGFR inhibitors; T790M evolution	Yes — initially T790M-negative DTPs survived	No — not a withdrawal-memory design	No.	Partial — evolutionary descendants tracked under treatment	Not central	Yes — early and late resistance had different dependencies	Yes — longitudinal clonal and single-cell-derived analyses	Separates early pre-existing T790M selection from later resistance through a persister reservoir
(Shaffer et al., 2017)	Melanoma; targeted therapy	Yes — rare pre-treatment states favored survival and reprogramming	Not central	No.	Partial — resistant descendants tracked under sustained selection	No.	Not central	Yes — single-cell state tracking	A pre-resistant state is not equivalent to post-treatment memory
(Hangauer et al., 2017)	Multiple lineages; BT474 breast cancer; lapatinib	Yes — persisters were GPX4-dependent	Yes — lapatinib removed; cells regrown up to 37 days	Yes — secondary vulnerability persisted transiently	Partial — prolonged regrowth; no division threshold	Partial — lapatinib re-exposure rederived persisters	Yes — GPX4-inhibitor sensitivity diminished with recovery	Not central	Original-drug sensitivity and a secondary vulnerability recovered on different timescales
(Rehman et al., 2021)	Colorectal cancer models; chemotherapy including irinotecan/FOLFIRI.	Yes — reversible diapause-like DTP state	Yes — treatment stopped; regrowth allowed	Yes — reversible regrowth	Partial — many barcoded clones traversed the state	Yes — regrown material retreated with CPT-11	Yes — autophagy inhibition reduced survival	Yes — cellular barcoding	Preserved barcode diversity does not prove retention of one molecular state
(Oren et al., 2021)	Multiple targeted-therapy lung and breast cancer models	Yes — cycling and non-cycling persisters differed	Not central	No.	No after withdrawal	No.	Yes — vulnerabilities differed by persister subgroup	Yes — Watermelon lineage/proliferation/single-cell profiling	Demonstrates heterogeneity during treatment, not post-withdrawal persistence
(Davern et al., 2026)	Selected drug-tolerant and immunotherapy-persister models	Yes — reduced mitochondrial apoptotic sensitivity	Not central	Not systematically tested	No.	Not central	Yes — selected cross-tolerance to drugs, radiation, or CAR-T cells	No longitudinal lineage tracing	Selected models do not establish universal cross-treatment tolerance
(Li et al., 2026b)	Melanoma/trametinib adaptation and reporter models	Yes — AP-1 promoted adaptation; CBP/p300 maintained reporter states	Yes — separate reporter experiment: 2 weeks trametinib, then 3 weeks DMSO.	Yes — reporter-state differences persisted	Partial — linked to resistant descendants; no universal division threshold	No complete trametinib withdrawal–rechallenge test	Not central	Yes — dual reporters, live-cell tracking, and barcoding	Supports molecular persistence, not complete functional memory
(Echeverria et al., 2019)	TNBC PDXs; doxorubicin plus cyclophosphamide	Yes — residual tumors changed state without major clonal selection	Yes — treatment stopped; tumors regrew	Yes, as decay — regrowth returned toward treatment-naive features	Partial — barcodes preserved clonal architecture; continuous state retention not shown	Yes — regrown tumors recovered chemotherapy sensitivity	Yes — OXPHOS inhibition delayed regrowth	Yes — barcoding and genomic sequencing	Strong reversible-tolerance evidence; no continuous molecular-state maintenance shown
(Chen et al., 2024)	Engineered persister models across cancer types	Yes — BET inhibition preferentially impaired cycling persister-derived cells	Yes — withdrawal generated cycling persister-derived cells; xenograft therapy switched at day 28	Yes — post-withdrawal outgrowth assessed	Partial — cycling persister-derived cells studied; no predefined division threshold	Yes — regrown cells recovered primary-drug sensitivity	Yes — BET inhibition delayed relapse	Not central	The vulnerability of cycling persister-derived cells should not be generalized to quiescent states
(Baudre et al., 2025)	Patient-derived TNBC models across treatments	Yes — shared stress/inflammatory programs; FOSL1/AP-1 promoted entry	Partial — treatment ceased before relapse; no standardized memory washout	Yes, as reversion — relapse returned toward treatment-naive transcriptomes	No division-resolved maintenance assay	No controlled original-agent rechallenge	Partial — FOSL1 loss reduced persister-state entry	Partial — patient-derived and single-cell analyses; no lineage recorder	Shared on-treatment programs and relapse-associated reversion do not prove continuous memory
(Liu et al., 2026)	EGFR-mutant lung cancer; PDO-CAF cultures and xenografts; osimertinib	Yes — RGS5+MYL9+ CAFs supported DTP survival	Yes — PDO washout and xenograft withdrawal	Yes — post-washout regrowth assessed	No division-resolved lineage assay	Partial — three-phase response assessed without lineage-resolved proof	Yes — fasudil blocked mitochondrial transfer and improved response	Partial — CAF single-cell analysis; no tumor-cell lineage recorder	Supports stromal contribution to survival/regrowth, not autonomous tumor-cell inheritance

This table compares representative studies across experimental dimensions and related design features. It is not a validated score, hierarchy, systematic review, or exhaustive evidence synthesis. The descriptive terms Yes, Partial, No, Not central, and Not systematically tested indicate only what each study assessed.

Abbreviations: CAF, cancer-associated fibroblast; CAR-T, chimeric antigen receptor T-cell; DTP, drug-tolerant persister; EGFR, epidermal growth factor receptor; GPX4, glutathione peroxidase 4; PDO, patient-derived organoid; PDX, patient-derived xenograft; TKI, tyrosine kinase inhibitor.

An EGFR-mutant lung adenocarcinoma patient-derived xenograft illustrates why tumor and stromal compartments should be followed together. Erlotinib-treated residual tumors preserved the baseline genomic clonal architecture but showed reduced proliferation and a single-cell transcriptional shift accompanied by replacement of the predominant fibroblast population with an IL-6-enriched state. This supports treatment-associated tumor-stroma adaptation under therapy, while the absence of a defined withdrawal-rechallenge sequence limits classification as post-treatment memory (Moghal et al., 2023).

## 5 Treatment strategies during DTP formation, residual disease, and regrowth

Interventions delivered with initial therapy primarily affect DTP formation, whereas treatment during residual disease is more likely to act on an established state. In the trametinib model, AP-1 participated in adaptation during low-dose exposure, while CBP/p300 remained involved in maintaining the reporter-gene state after its formation, suggesting that early and late phases of the same treatment course may not share identical dependencies (Li et al., 2026b). Epigenetic and stress-response pathways also contribute broadly to normal tissue homeostasis, and additional drugs may increase toxicity. A combination that improves early cell killing alone does not establish that it alters the state remaining after withdrawal.

The residual-disease phase provides an experimental setting in which DTP-directed interventions have been evaluated. Dependencies on GPX4, ferroptosis defense, autophagy, antioxidant metabolism, fatty acid oxidation, and anti-apoptotic mechanisms have each been therapeutically exploitable in specific models (Hangauer et al., 2017; Oren et al., 2021; Rehman et al., 2021; Wang et al., 2021; Davern et al., 2026). These dependencies do not coexist in every DTP. An intervention that eliminates only quiescent cells may leave behind persisters that continue to divide. These observations support testing state-matched interventions against functionally characterized residual populations.

During drug holidays or intermittent therapy, reversible states may regain sensitivity to the original agent while residual cells continue to expand. Preclinical observations raise the possibility that a rechallenge window may exist when recovery of sensitivity precedes substantial residual-cell regrowth; if regrowth is faster, withdrawal may instead shorten disease control. The EGFR model further suggests that early expansion of pre-existing mutant clones and later DTP-derived resistance may require different experimental timing of combination treatment (Hata et al., 2016). These considerations provide a rationale for prospectively testing treatment timing, not for routine treatment interruption or rechallenge in current clinical practice. Figure 1B illustrates this preclinical competing-timescale hypothesis.

Strategies that deepen the initial response should be distinguished from interventions that erase an established post-treatment state. In lung adenocarcinoma models, restoring ZNF263 reduced osimertinib persistence and tumor regrowth, whereas Aurora B inhibition enhanced BIM- and PUMA-mediated apoptosis and reduced emergence of tolerant cells during EGFR inhibition. Both approaches target survival during therapy; whether either shortens the lifetime of a state after withdrawal requires separate testing (Tanaka et al., 2021; Liang et al., 2024).

## 6 Clinical validation of markers of treatment history

Clinical identification of a persistent treatment-associated state requires longitudinal evidence rather than a single post-treatment marker. An informative sequence would combine pretreatment tissue, sampling during early therapy and minimal residual disease, confirmation of pharmacologic clearance after treatment completion or a clinically indicated interruption, state and clonal characterization, and a subsequent functional or clinical outcome. A candidate marker would be more informative if it remains detectable after drug clearance, can be assigned to the same lineage or clonal descendants, and is associated with response to later therapy or relapse in an independent cohort. Figure 1D summarizes this translational validation sequence and its principal boundary: an association with later response or relapse is required, whereas a single post-treatment marker is insufficient.

Circulating tumor DNA can track genetic clones and the emergence of stable resistance alterations, but it cannot independently demonstrate a reversible cell-state memory. Circulating tumor cells, exosomes, and imaging may complement tissue analysis, although none currently substitutes for paired state measurements and a functional consequence. Patient-derived organoids and xenografts can bridge clinical sampling to controlled withdrawal and rechallenge experiments, but they cannot replace longitudinal evidence in patients. Prospective studies should use samples obtained after treatment completion or clinically indicated interruption; treatment should not be withheld solely to create a washout interval.

Continuous high-dose exposure *in vitro* differs from intermittent exposure and pharmacokinetic fluctuation in patients. DTP studies should therefore report treatment dose and duration, residual fraction, withdrawal and clearance conditions, elapsed time and passage number, rechallenge design, clonal complexity, and state reversibility (Russo et al., 2024; Lopez-Gonzalez et al., 2026). These variables are needed to determine whether a marker represents acute stress, persistent state retention, clonal selection, or stable resistance.

## 7 Tumor microenvironment and residual-state persistence

Microenvironmental support can influence both the abundance and composition of residual cells (Russo et al., 2024). In an EGFR-mutant lung adenocarcinoma xenograft, erlotinib-treated residual tumors retained baseline clonal architecture but acquired an IL-6-enriched fibroblast state (Moghal et al., 2023). A separate study identified RGS5+MYL9+ cancer-associated fibroblasts that received damaged mitochondria from TKI-exposed tumor cells through tunneling nanotubes; these fibroblasts improved DTP survival and post-washout regrowth in organoid and xenograft models (Liu et al., 2026). These findings support stromal contributions to treatment-period persistence and regrowth, but they do not establish that a tumor-cell-intrinsic memory state is maintained independently across divisions.

Hypoxia and spatial organization add further heterogeneity. A single-cell atlas of residual triple-negative breast cancer xenografts after neoadjuvant chemotherapy identified cycling and non-cycling hypoxia-related persister states and validated related programs in patient cohorts; because some programs were already detectable before treatment, selection and induction remain difficult to separate (Zhang et al., 2026). In a melanoma xenograft studied during sustained BRAF/MEK inhibition, lineage and spatial analyses identified persister-associated stress, lipid-metabolism, PI3K-signaling, and extracellular-matrix-remodeling programs, together with inferred autocrine and paracrine interactions (Li et al., 2026a). Both studies characterize residual disease under or after therapy, but neither provides a complete withdrawal-lineage-rechallenge demonstration.

Immune-cell composition may also be shaped by persister populations. In a humanized EGFR-mutant lung adenocarcinoma model, DTP-derived CCL20 was associated with an immunosuppressive microenvironment; combined CCL20 and PD-1 blockade altered immune-cell composition and reduced tumor growth (Kwok et al., 2026). This experiment supports bidirectional coupling between persister cells and immune context during treatment, but it did not test maintenance after a defined withdrawal interval.

Microenvironmental inputs remain context-dependent candidates for sustaining residual states. To establish a role after treatment withdrawal, co-culture or *in vivo* studies need to confirm drug clearance, follow tumor and host compartments through the drug-free interval, and test whether disrupting the niche changes state persistence and the response to rechallenge.

## 8 Limitations of the current evidence

Current studies do not use a standardized definition or threshold for post-treatment cellular memory. Drug concentration, exposure duration, washout procedures, elapsed time, passage number, and functional endpoints vary substantially, and the same number of drug-free days can represent very different numbers of cell divisions (Russo et al., 2024; Lopez-Gonzalez et al., 2026). Residual drug, selection of a pre-existing clone, culture drift, and stable genetic resistance can each mimic persistence unless they are addressed directly.

Few studies combine confirmed withdrawal, longitudinal state measurements, lineage or clonal analysis, and functional rechallenge in the same model. Serial human samples are particularly limited, and clinically indicated interruptions rarely reproduce controlled experimental washout. This narrative review did not apply formal risk-of-bias assessment or exhaustive screening, so its evidence comparisons are intended to clarify experimental interpretation rather than estimate the prevalence of post-treatment memory across cancers.

## 9 Conclusion

Evidence is strongest for the existence and heterogeneity of DTP states during treatment. Selected studies additionally demonstrate recovery of drug sensitivity after withdrawal, persistence of molecular states or secondary vulnerabilities for a limited interval, or altered responses to later treatments. Far fewer studies combine confirmed drug clearance, longitudinal measurement across clonal descendants, lineage information, and functional rechallenge in the same model. Post-treatment cellular memory is therefore best used as an operational description of treatment-associated state retention whose strength depends on the experimental dimensions actually tested. Separating early state formation from post-withdrawal maintenance, and pre-existing resistant clones from resistance emerging through a persister reservoir, identifies the experiments needed to connect residual-cell biology with treatment timing. The resulting scheduling and rechallenge hypotheses require prospective testing and do not constitute an established clinical strategy.
