# Supplementary material for: Cellular memory after cancer therapy: persistence after drug withdrawal, maintenance across cell divisions, and responses to rechallenge
Source: Front Pharmacol. 2026 Oct 5;17:1966584. doi: 10.3389/fphar.2026.1966584 (PMC13678069; doi:10.3389/fphar.2026.1966584)
Supplement: Supplementary file 1 [file Table1.docx]

Supplementary Table S1. Extended evidence matrix of representative experimental studies informing post-treatment cellular memory

| **Study, year, and reference** | **Cancer model and initial treatment** | **Residual phenotype and principal mechanism** | **Withdrawal or washout design** | **Post-withdrawal duration and divisions/clonal descendants** | **Original-treatment rechallenge** | **Secondary vulnerability or cross-treatment response** | **Lineage-tracing or single-cell approach** | **Main contribution and critical evidence boundary** |
| --- | --- | --- | --- | --- | --- | --- | --- | --- |
| Sharma et al., 2010 | Several cancer cell lines; PC9 EGFR-mutant lung cancer cells highlighted; prolonged targeted-drug exposure. | Rare reversible DTP population; approximately 0.3% of starting PC9 cells; >100-fold reduction in sensitivity; IGF-1R and KDM5A-associated chromatin regulation. | Yes; selected cells were removed from drug and allowed to regrow. | Regrowth after withdrawal was demonstrated; the tolerant phenotype could be entered and exited at low frequency. | Regrown cells recovered sensitivity to the original agent. | Not a principal endpoint. | No formal lineage-tracing platform in the evidence summarized here. | Establishes that survival during treatment is not equivalent to post-treatment memory. Recovery of drug sensitivity supports reversibility. |
| Hata et al., 2016 | EGFR-mutant lung cancer models treated with EGFR inhibitors. | Early resistance from pre-existing T790M-positive clones and later T790M-positive resistance emerging from initially T790M-negative DTPs. | No defined drug-free washout designed to test memory. | Longitudinal evolution under treatment; early and late resistant populations arose on different timelines. | Not a complete withdrawal-rechallenge design. | Late resistant cells had distinct apoptotic dependencies and therapeutic vulnerabilities. | Longitudinal clonal, genotypic, and single-cell-derived analyses. | Shows that DTP survival can extend the period available for genetic evolution. It does not prove direct induction of T790M by a non-genetic state. |
| Shaffer et al., 2017 | Melanoma models exposed to targeted therapy. | Rare pre-treatment cells transiently expressed resistance-associated genes; drug exposure promoted epigenetic and transcriptional reprogramming toward stable resistance. | No defined withdrawal design central to the study. | Progressive reprogramming and later stable resistance were followed over time, but post-withdrawal divisions were not the defining endpoint. | Not explicitly tested as withdrawal followed by original-drug rechallenge. | Not a principal endpoint. | Single-cell expression measurements and tracking of rare cellular states. | Supports combined pre-existing variability and treatment-induced reprogramming. A pre-resistant state is not equivalent to stable resistance or post-treatment memory. |
| Hangauer et al., 2017 | Several cancer lineages; BT474 breast cancer cells treated with lapatinib were a key model. | Persisters were vulnerable to GPX4 inhibition and ferroptosis. | Yes; BT474 persisters were generated by 9 days of lapatinib, removed from drug, and regrown for 37 days. | Regrown cells were subsequently re-exposed to lapatinib to generate rederived persisters. | Re-exposure to lapatinib re-established the persister context; the study also assessed recovery from the initial state. | Sensitivity to GPX4 inhibitors such as RSL3/ML210; vulnerability could diminish with recovery. | No formal lineage-tracing approach was central. | Supports persistence or re-establishment of a secondary vulnerability on a different timescale from original-drug sensitivity. Not universal across all DTPs. |
| Rambow et al., 2018 | BRAF-mutant melanoma patient-derived xenografts treated with RAF/MEK inhibitors. | Multiple interconvertible MRD states, including an RXR-dependent neural crest-like state. | No defined drug-free washout; focus was MRD under therapy. | State interconversion and spatial heterogeneity were assessed within residual disease. | Original-agent rechallenge after withdrawal was not the primary test. | RXR-pathway inhibition reduced accumulation of the neural crest-like state and delayed resistance. | Single-cell RNA sequencing and spatially informed analysis. | Demonstrates heterogeneity and plasticity in residual lesions. Coexisting states during treatment do not by themselves establish post-withdrawal memory. |
| Rehman et al., 2021 | Colorectal cancer cell and patient-derived models treated with chemotherapy, including irinotecan/FOLFIRI contexts. | Reversible embryonic diapause-like DTP state with reduced proliferation and autophagy dependence. | Treatment was stopped to permit tumor regrowth. | Regrown tumors retained substantial barcode complexity, suggesting that many clones traversed the DTP state. | Regrown material was reinjected and retreated with CPT-11. | Autophagy inhibition reduced persister survival. | Cellular barcoding and population analyses. | Supports broad, reversible entry into a DTP state and preserved clonal complexity. Barcode persistence does not prove persistence of a specific molecular state. |
| Oren et al., 2021 | Multiple targeted-therapy models, including EGFR-mutant lung and breast cancer systems. | Cycling and non-cycling persisters arose from distinct lineages; cycling persisters showed stronger antioxidant and FAO programs. | No standardized withdrawal interval was central to the analysis. | Clonal origin, proliferative state, and transcriptional programs were measured during treatment. | Original-agent rechallenge after withdrawal was not the primary endpoint. | Metabolic vulnerabilities differed between persister subpopulations. | Watermelon barcoding integrated lineage, proliferation, and single-cell transcriptional information. | Demonstrates lineage and metabolic heterogeneity under treatment. Does not establish that all identified states persist after withdrawal. |
| Wang et al., 2021 | Platinum-tolerant ovarian cancer models. | FZD7-marked persister cells showed a defined ferroptosis vulnerability. | No drug-free washout designed to test memory was central to the study. | Post-withdrawal persistence across divisions was not established. | Original-platinum rechallenge was not the defining endpoint. | Ferroptosis sensitivity in the FZD7-positive residual population. | No formal lineage-tracing approach was central. | Identifies an exploitable vulnerability in a defined ovarian-cancer persister population. It should not be treated as a universal DTP marker. |
| Radic Shechter et al., 2021 | Residual breast cancer lesions after treatment. | Persistent metabolic alterations associated with minimal residual disease. | Persistence after treatment cessation was described, but a standardized washout design was not central. | A metabolic signature remained for a post-treatment interval; exact division-based maintenance was not established. | Original-treatment rechallenge was not used to establish functional memory. | Metabolic dependencies were identified in residual disease. | Multi-omic/metabolic profiling rather than formal lineage tracing. | Indicates that metabolic states can persist after treatment. Molecular persistence alone does not establish altered rechallenge response. |
| Davern et al., 2026 | Selected drug-tolerant and immunotherapy-persister models. | Reduced mitochondrial apoptotic sensitivity across selected persister states. | A defined washout and division-based persistence design was not the principal focus. | Post-withdrawal maintenance was not systematically established. | The original-agent rechallenge was not the sole focus. | Some populations showed cross-resistance to drugs, radiotherapy, or CAR-T-cell attack. | No longitudinal lineage-tracing design was central. | Supports testing responses beyond the original drug. Findings from selected models do not establish universal cross-treatment tolerance. |
| Li et al., 2026 | Melanoma/trametinib adaptation models; reporter experiments included transient dexamethasone induction. | AP-1 contributed to adaptation; CBP/p300 acetyltransferase activity and bromodomain function maintained established reporter states. | In a separate reporter experiment, 2 weeks of trametinib were followed by 3 weeks in DMSO. | Reporter-state differences remained detectable after withdrawal and were linked to resistant-clone descendants. | No complete withdrawal followed by original-trametinib rechallenge demonstrating altered function. | Not a principal endpoint. | Dual-reporter live-cell tracking and DNA barcoding. | Supports molecular-state persistence and distinct establishment versus maintenance mechanisms. Does not satisfy the full functional memory definition without rechallenge. |
| Liu et al., 2024 | Pan-cancer transcriptomic analysis across chemotherapy-treated tumor models. | Shared residual-disease transcriptional programs, including DUSP1-associated chemotherapy persistence. | A standardized drug-free washout was not central across models. | Persistence across divisions or clonal descendants was not uniformly established. | Original-treatment rechallenge was not systematically assessed across models. | Not systematically tested. | Cross-model transcriptomic analysis rather than lineage tracing. | Supports the possibility of shared residual-state programs. Shared expression does not prove common lineage origin, withdrawal persistence, or a common rechallenge phenotype. |
| Ramirez et al., 2016 | EGFR-addicted lung cancer; erlotinib. | A single-cell-derived persister bottleneck generated colonies with diverse stable resistance mechanisms. | Not a defined washout-memory design. | Longitudinal emergence of resistant colonies from a persister ancestor. | Not the principal endpoint. | Diverse drug-response profiles among resistant colonies. | Single-cell origin, drug screening, and whole-exome sequencing. | Shows that a persister reservoir can seed heterogeneous resistance; it does not define a single deterministic route. |
| Echeverria et al., 2019 | TNBC PDX models; doxorubicin plus cyclophosphamide; serial patient biopsies. | Reversible residual state with molecular and histologic changes but stable subclonal architecture. | Treatment was stopped and residual tumors were allowed to regrow. | Regrown tumors returned toward treatment-naive-like molecular features. | Regrown tumors recovered chemotherapy sensitivity. | OXPHOS inhibition delayed residual-tumor regrowth. | Barcode clonal tracking and genomic sequencing. | Directly supports reversible tolerance without major clonal selection; continuous state retention after withdrawal was not shown. |
| Kurppa et al., 2020 | EGFR-mutant lung cancer; combined EGFR and MEK inhibition. | Senescence-like dormancy driven by YAP/TEAD-SLUG repression of BMF. | No defined washout central to the design. | Not established. | Not a withdrawal-rechallenge study. | YAP/TEAD perturbation depleted dormant cells by enhancing apoptosis. | Genetic and pharmacologic causal perturbation. | Defines an on-treatment survival mechanism, not post-withdrawal memory. |
| Kashima et al., 2021 | EGFR-mutant lung cancer cell models and clinical specimens; EGFR TKIs. | Diverse tolerant-to-resistant trajectories; CD74 contributed to survival and regrowth. | Yes — xenografts received osimertinib for 11 or 28 days, followed by a defined 14-day withdrawal. | Tumor regrowth was measured during the 14-day withdrawal; division-resolved maintenance was not assessed. | Not assessed as withdrawal followed by original-agent rechallenge. | CD74 upregulation blocked apoptosis and supported regrowth. | Single-cell RNA-seq and ATAC-seq with functional validation. | Resolves heterogeneity but does not prove that every state persists after withdrawal. |
| Moghal et al., 2023 | EGFR-mutant lung adenocarcinoma PDX; erlotinib for 5-6 weeks. | Low-proliferation DTP tumors with NF-kB/STAT3-associated programs and IL-6-enriched fibroblasts. | Residual disease was assessed during treatment. | Post-withdrawal duration was not established. | Not tested as a complete withdrawal-rechallenge sequence. | Tumor-stroma adaptation was implicated in survival. | Whole-exome, bulk RNA, and single-cell RNA sequencing. | Preserved clonal architecture does not by itself establish post-withdrawal memory. |
| Pfeifer et al., 2024 | EGFR-mutant lung cancer cells and patient-derived organoids; osimertinib or gefitinib. | Hippo/YAP-TEAD signaling promoted persister survival. | No defined washout central to the study. | Not established. | Not a withdrawal-rechallenge study. | Combined EGFR and Hippo-pathway targeting reduced persister survival. | Genome-wide CRISPR knockout and activation screens with validation. | Supports a model-specific on-treatment dependency, not universal memory maintenance. |
| Haderk et al., 2024 | Patient-derived, immunocompetent, and clinical lung cancer systems under EGFR-, ALK-, or KRAS-targeted therapy. | FAK-YAP signaling promoted residual DTP survival. | Focus was residual disease under treatment. | Not established after drug withdrawal. | Not the principal endpoint. | FAK-YAP inhibition with targeted therapy suppressed residual disease. | Integrated preclinical models and clinical specimens. | Cross-model recurrence of a pathway does not establish post-withdrawal persistence or universal dependence. |
| Chen et al., 2024 | Engineered DTP models across multiple cancer types. | BET inhibition selectively harmed cycling persister-derived cells by suppressing redox regulators. | Yes — drug withdrawal generated proliferating persister-derived cells; in vivo targeted therapy was switched at day 28 to vehicle or BET inhibitor. | Post-withdrawal persister-derived outgrowth was assessed; divisions were not quantified by a predefined threshold. | Yes — regrown cells recovered sensitivity to the primary targeted therapy. | BET inhibition delayed melanoma and lung cancer relapse in vivo. | Functional genetic and compound screens. | A vulnerability of cycling persisters should not be generalized to quiescent or unrelated residual states. |
| Sun et al., 2025 | EGFR- or KRASG12C-mutant lung cancer; matched targeted inhibitors. | PRMT1 supported persistence in STAT1-high models. | No defined drug-free memory interval central to the study. | Regrowth delay was measured after combination treatment. | Not the defining endpoint. | PRMT1 knockdown reduced persistence and delayed regrowth; chromosome 5q31.1 status may stratify response. | Isoform-specific genetic perturbation and xenografts. | Effect was genotype/context dependent and concerns persistence during treatment. |
| Liang et al., 2024 | Lung adenocarcinoma models and clinical residual tumors; osimertinib. | ZNF263 loss marked resistant and persister states; restoration altered EGFR regulation and reduced persistence. | Yes — xenografts received osimertinib for 10 days followed by a defined 10-day withdrawal; in vitro DTPs were generated during 10 days of continued exposure. | Tumor regrowth was measured during the 10-day withdrawal; divisions or clonal descendants were not resolved. | Not assessed as withdrawal followed by original-agent rechallenge. | ZNF263 restoration enhanced EGFR-targeted response. | Molecular perturbation and xenograft validation. | Supports prevention or reduction of persister formation rather than erasure of established memory. |
| Kawakami et al., 2020 | Patient-derived gastric cancer models; 5-FU or SN38. | ALDH1A3-high residual cells engaged an mTOR-associated survival program. | Yes — for single-cell expression analysis, 7 days of 5-FU were followed by 4 days without drug to reduce transient drug-induced expression. | Residual-cell expression was assessed after 4 drug-free days; division-resolved maintenance was not assessed. | Not assessed as withdrawal followed by original-agent rechallenge. | ALDH1A3 depletion or temsirolimus reduced persister abundance. | Single-cell lineage-related expression analysis and functional screening. | Defines a gastric-cancer-specific marker and dependency, not a universal DTP marker. |
| Wu et al., 2024 | Head and neck cancer DTP models; cisplatin; patient-derived and xenograft contexts. | FSP1 and lipid/iron-homeostasis programs were linked to tolerance and ferroptosis regulation. | No defined drug-free interval central to the study. | Not established across divisions. | Original-cisplatin rechallenge was not the defining endpoint. | FSP1 targeting promoted ferroptotic vulnerability in preclinical models. | Transcriptomic, perturbation, and xenograft analyses. | Model-specific ferroptosis regulation should not be generalized to all DTP states. |
| Tanaka et al., 2021 | EGFR-mutant lung cancer; osimertinib and Aurora B inhibition. | Aurora B blockade enhanced BIM- and PUMA-mediated apoptosis and countered EMT-associated resistance. | No defined post-withdrawal memory design. | Not established. | Not the principal endpoint. | Combination treatment reduced emergence of tolerant cells and overcame selected resistant states. | High-throughput compound screening and mechanistic validation. | Improved initial killing is not evidence of altered state persistence after withdrawal. |
| Garcia et al., 2025 | EGFR-mutant lung cancer; gefitinib/osimertinib contexts. | APOBEC3 activity promoted DTP survival, T790M acquisition, and squamous transdifferentiation. | Evolution was followed under treatment rather than a defined washout. | DTP-to-resistant evolution was assessed. | Not the defining endpoint. | p63 perturbation altered resistant-cell sensitivity to osimertinib. | Genetic perturbation and evolutionary analyses. | Shows one source of diversification; it does not imply inevitable APOBEC-driven evolution. |
| Punzi et al., 2025 | Human cancer cells exposed to FOLFOX, FOLFIRI, and related chemotherapy regimens. | Early autophagy-associated tolerance transitioned to later persistence with altered DNA-repair regulation involving PINK1/HNF4A. | Time-resolved exposure was central; a drug-free memory interval was not the primary endpoint. | Barcodes and temporal profiling distinguished early and late surviving populations. | Not a complete withdrawal-rechallenge sequence. | Stage-specific autophagy/mitophagy and DNA-repair programs suggested temporal vulnerabilities. | Temporal multi-omic and barcode analyses. | Tolerance and persistence were separated during exposure; this does not establish post-withdrawal functional memory. |
| Joun et al., 2025 | Glioblastoma stem-cell models under chemotherapy. | PRDM9-dependent H3K4me3 enhanced cholesterol biosynthesis and oxidative-stress tolerance. | Yes — DTPs generated during 14 days of microtubule-targeting-agent exposure were placed in drug-free medium; recovery assays used 7–21 days depending on cell line. | Surviving cells resumed proliferation and recolonized within approximately 14 days; no predefined division threshold was used. | Yes — recovered cells were rechallenged with CMPD1 or tivantinib and yielded drug-sensitive progeny. | PRDM9 inhibition with chemotherapy improved the magnitude and duration of preclinical response. | Histone proteomics, transcriptomics, lipidomics, ChIP-seq, CRISPR, and phenotypic screening. | Supports a glioblastoma-specific survival program and reversible drug tolerance; it does not establish a predefined division-based memory threshold. |
| Baudre et al., 2026 | Multiple patient-derived TNBC models across therapies, with comparison to HER2-positive breast and lung cancer systems. | Basal-keratin, stress, and inflammatory programs converged in persisters; FOSL1/AP-1 promoted entry. | Partial — treatment was stopped and tumors were followed to relapse; no standardized drug-free interval was specified as a memory assay. | At relapse, transcriptomes reset toward a treatment-naive-like state; division-resolved maintenance was not assessed. | Not assessed as a controlled original-treatment rechallenge. | FOSL1 loss reduced entry into the persister state. | Patient-derived models, transcriptomics, regulatory-network inference, and functional perturbation. | Shared expression programs do not prove a shared lineage origin or identical withdrawal-rechallenge phenotype. |
| Liu et al., 2026 | EGFR-mutant lung cancer cells, matched PDO-CAF cultures, xenografts, and patient tissue; osimertinib. | RGS5+MYL9+ CAFs accepted damaged tumor mitochondria through tunneling nanotubes and supported DTP survival. | PDOs underwent washout after 72 hours; xenografts received osimertinib for 9 days followed by withdrawal. | Post-washout regrowth was measured; division-resolved maintenance and tumor-cell lineage tracing were not central. | Partially assessed in a three-phase model; not a lineage-resolved proof of autonomous memory. | Fasudil blocked mitochondrial transfer and improved osimertinib response in vivo. | Single-cell RNA sequencing characterized CAFs; no tumor-cell lineage recorder was central. | Supports a stromal contribution to DTP survival and post-withdrawal regrowth, not autonomous inheritance of a tumor-cell state. |
| Kwok et al., 2026 | H1975 EGFR-mutant lung adenocarcinoma in humanized NSG mice; anti-PD-1 and anti-CCL20. | DTP-derived CCL20 was associated with an immunosuppressive tumor immune microenvironment. | Not tested. | Not tested after withdrawal; divisions or clonal descendants were not assessed. | Not tested as original-agent rechallenge. | Combined CCL20 and PD-1 blockade altered immune-cell composition and reduced tumor growth. | Single-cell RNA sequencing and multiplex immunofluorescence; no longitudinal tumor-cell lineage tracing. | Supports immune coupling to DTPs during treatment in one model; it does not establish post-withdrawal maintenance. |
| Zhang et al., 2026 | Fourteen TNBC PDX models with residual disease after neoadjuvant chemotherapy; independent patient cohorts. | Hypoxia-related cycling and non-cycling persister states were identified after therapy. | Treatment completion was represented, but a standardized pharmacologic washout was not the central design. | Related programs were observed in metastatic disease; no division-resolved lineage-maintenance assay was performed. | Not tested. | KDM5B inhibition suppressed emergence of DTPs in vitro. | Single-cell profiling and cohort validation; formal lineage tracing was not central. | Supports hypoxia-associated heterogeneity in residual disease, but not a complete withdrawal-lineage-rechallenge sequence. |
| Li et al., 2026 | Patient-derived melanoma PDX during prolonged BRAF/MEK inhibition. | Four persister-associated states included stress, lipid metabolism, PI3K signaling, and ECM remodeling. | Not tested; treatment was sustained through MRD and recurrence. | Not tested after withdrawal; clonal descendants were tracked only under treatment. | Not tested. | Autocrine and paracrine interactions were inferred from spatial transcriptomic analyses. | MeRLin barcoding, single-cell RNA sequencing, RNA-FISH, and spatial transcriptomics. | Supports spatially organized, lineage-resolved heterogeneity during treatment; inferred cell-cell communication is not post-withdrawal memory. |

Note: This narrative evidence matrix compares study designs across treatment, withdrawal, post-withdrawal persistence, descendants, rechallenge, secondary response, and lineage evidence. It is not a validated scoring system or exhaustive synthesis. NR should be used only when the original publication does not report the relevant element.

**Abbreviations:** CAR-T, chimeric antigen receptor T-cell therapy; DTP, drug-tolerant persister; EGFR, epidermal growth factor receptor; FAO, fatty-acid oxidation; FZD7, frizzled class receptor 7; GPX4, glutathione peroxidase 4; IGF-1R, insulin-like growth factor 1 receptor; KDM5A, lysine-specific demethylase 5A; MRD, minimal residual disease; RXR, retinoid X receptor.
